# Tolerance and Long-Term MRI Imaging of Gadolinium-Modified Meshes Used in Soft Organ Repair

**DOI:** 10.1371/journal.pone.0120218

**Published:** 2015-03-26

**Authors:** Vincent Letouzey, Stéphanie Huberlant, Arnaud Cornille, Sébastien Blanquer, Olivier Guillaume, Laurent Lemaire, Xavier Garric, Renaud de Tayrac

**Affiliations:** 1 Department of Obstetrics and Gynecology, Nîmes University Hospital, Place du Pr R. Debré, 30000, Nîmes, France; 2 Department of Artificial Polymers, Max Mousseron Institute of Biomolecules, CNRS UMR 5247, 34000, Montpellier, France; 3 Institut National de la Santé et de la Recherche Médicale, UMR-S 1066, MINT, 49000, Angers, France; National Institute of Radiological Sciences, JAPAN

## Abstract

**Background:**

Synthetic meshes are frequently used to reinforce soft tissues. The aim of this translational study is to evaluate tolerance and long-term MRI visibility of two recently developed Gadolinium-modified meshes in a rat animal model.

**Materials and Methods:**

Gadolinium-poly-ε-caprolactone (Gd-PCL) and Gadolinium-polymethylacrylate (Gd-PMA) modified meshes were implanted in Wistar rats and their tolerance was assessed daily. Inflammation and biocompatibility of the implants were assessed by histology and immunohistochemistry after 30 days post implantation. Implants were visualised by 7T and 3T MRI at day 30 and at day 90. Diffusion of Gadolinium in the tissues of the implanted animals was assessed by Inductively Coupled Plasma Mass Spectrometry.

**Results:**

Overall Gd-PMA coated implants were better tolerated as compared to those coated with Gd-PCL. In fact, Gd-PMA implants were characterised by a high ratio collagen I/III and good vascularisation of the integration tissues. High resolution images of the coated mesh were obtained *in vivo* with experimental 7T as well as 3T clinical MRI. Mass spectrometry analyses showed that levels of Gadolinium in animals implanted with coated mesh were similar to those of the control group.

**Conclusions:**

Meshes coated with Gd-PMA are better tolerated as compared to those coated with Gd-PCL as no signs of erosion or significant inflammation were detected at 30 days post implantation. Also, Gd-PMA coated meshes were clearly visualised with both 7T and 3T MRI devices. This new technique of mesh optimisation may represent a valuable tool in soft tissue repair and management.

## Introduction

Synthetic meshes have been routinely used for soft tissue reinforcement since their introduction in late 1950s [1]. Although hernia and abdominal wall repair is the most common application, reconstructive surgery and in particular pelvic organ prolapse (POP) repair are practiced in a large number of patients with success rates going up to 90% for the cystocele repair [2,3].

Meshes can be classified according to their properties including pore size and fiber type [4], weight and biomaterial composition [5].

Those commonly used in clinical practice are made of: (i) macroporous polypropylene (PP), which is a non-absorbable material; (ii) polyester (PET); polytetrafluoroethylene (PTFE) and expanded PTFE, etc. [6, 7]. Randomised prospective trials have underlined the interest of the treatment by synthetic meshes in terms of quality, low postoperative morbidity and long-term results, balanced with the recurrence of surgery due to mesh complication [8,9].

Indeed, for POP repair a prevalence of 15 to 20% of short-medium-term mesh complications (more than 6 months) is reported. These include pain, discomfort, vaginal bleeding, abnormal discharge, and recurrence of the prolapse that may eventually necessitate implant removal [10,11]. For hernia and abdominal wall repair common complications are chronic pain, seroma and adhesions [12,13].

Therefore, it is essential to optimise the mesh in order to improve tolerance and biocompatibility to the implant. Also, to improve management of complications and reduce recurrence of surgery, monitoring the position or a possible shrinkage of the implants would be a considerable advantage. Also, the possibility to visualise the mesh may be helpful for its removal following a complication. Magnetic resonance imaging (MRI) seems to be the most powerful imaging technique for the pelvic exploration [14,15]. Unfortunately, PP meshes are not spontaneously visible in MRI and the interposition of fascia makes the demarcation of implants impossible [16]. We previously described a method to covalently bind gadolinium to resorbable (poly(ε-caprolactone), PCL) and non-resorbable (poly(methyl acrylate), PMA) polymers and then coat the resulting Gd-polymers onto PP meshes. With this approach we obtained a satisfactory visualisation of the optimised meshes in experimental (*in vitro*) and clinical (*in vivo*) MRI [17]. With the present study we sought to evaluate (i) the preclinical tolerance and (ii) long-term MRI visibility of the previously described Gadolinium-modified PP meshes.

To this purpose we used a rat animal model, which is one of the preferred animal model for mesh fascia repair research [18–20] and two distinct method of implantation: (i) within the abdominal wall to assess the tolerance and biocompatibility and (ii) dorsal implantation to evaluate MR imaging.

## Materials and Methods

### Animals and surgical procedure and evaluation of clinical tolerance

*In vivo* experiments were performed at Nîmes University Hospital. The study was approved by the appropriate Animal Research Ethics Committee (CEEA-LR-1010) and conducted according to European Union animal care guidelines (Directive 2010/63/EU). All rats were female, 9–10 weeks old and weighed more than 300 g (mean 355 g ± 40g). The animals were housed on a 12:12-hour light–dark cycle and food and water were available ad libitum. All animals were anaesthetised with 2.5% halothane (0.5 L/min) and ketamine (50 mg/kg) and all efforts were made to minimise suffering.

**Fig 1:**
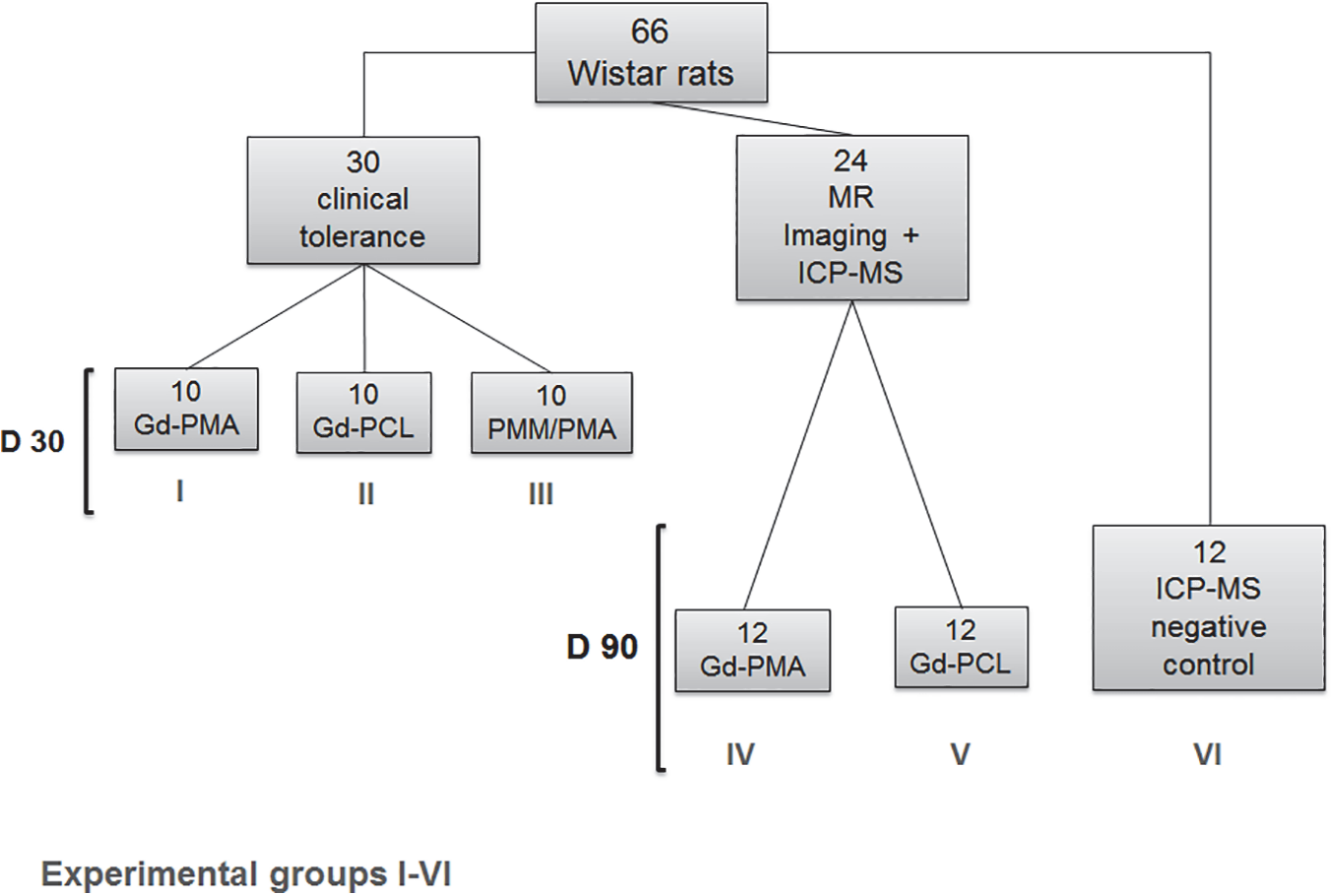
Flowchart showing experimental and control groups.

**Fig 2:**
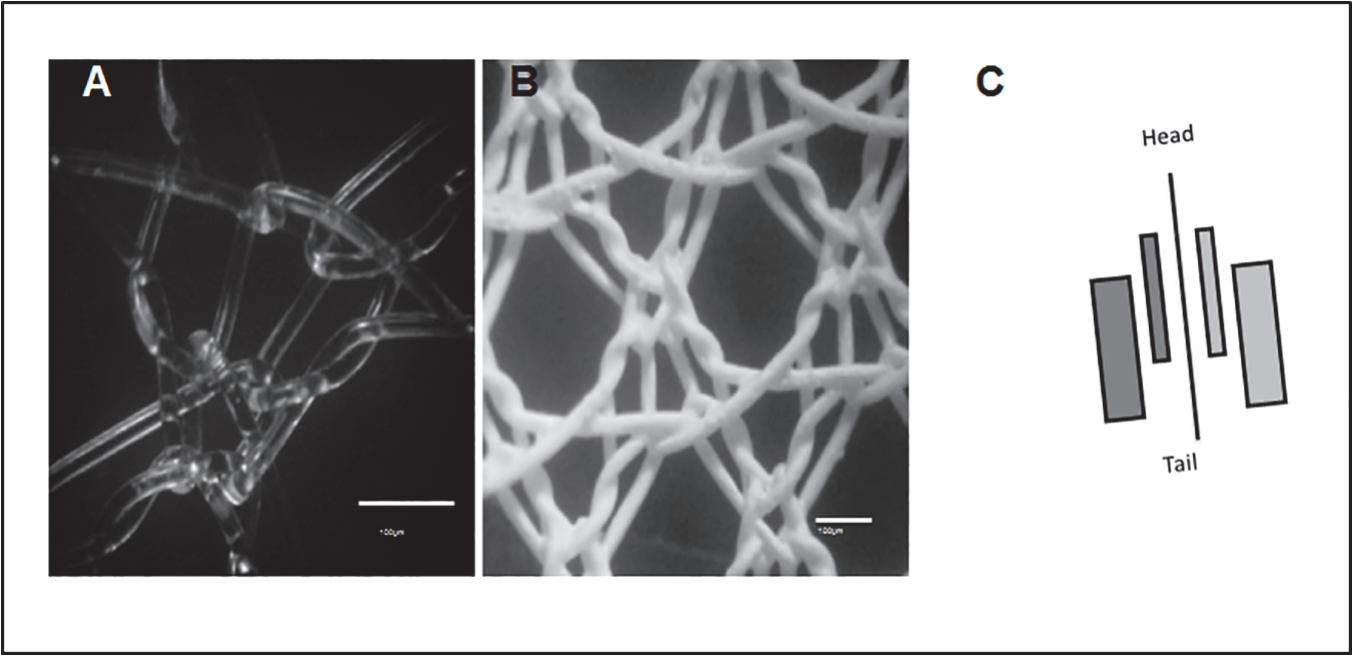
Microscopic view of the mesh. (A) Mesh before coating; (B) Gd-PMA Coated mesh as observed at an optical microscope (X100). (C) Schematic view of the implantation sites in rat: Gd-PMA coated mesh on the left (purple) and PMA/PMMA mesh (uncoated control) on the right side (blue).

A total of 66 Wistar rats (Charles River Laboratoires, L'Arbresle, France) were used during this study; all rats were randomised to one of the 6 groups of the study (see Fig. 1). For the tolerance experiments 30 rats were used: two test groups (Gd-PMA- experimental group I; and Gd-PCL experimental group II-modified meshes) and a control group (PMA/PMMA (poly(methyl methacrylate)) modified meshes—control group III) were analysed with ten rats allocated to each group; all meshes were implanted within the abdominal wall. The surgical procedure used is based on incisional abdominal hernia and has been previously described [21]. For MR imaging two test groups were analysed: Gd-PMA (experimental group IV) 12 rats and Gd-PCL (experimental group V) 12 rats. In each rat four meshes were implanted, two coated with Gd-complexes and two coated with PMA/PMMA. To this purpose subcutaneous dorsal implantations were performed: two 2-cm incisions were made on each side of the backbone, two pieces of Gd-complex mesh of 20 x 30 mm and 10 x 30 mm in size were implanted on the lumbar muscle on the left side and sutured. PMA/PMMA control meshes were similarly implanted but on the right side and sutured (Fig. 2). For both pairs, the meshes were placed at a millimetre distance. All animals that underwent surgery were assessed for signs of local (erosions) or systemic complications (infection) every day.

MRI analyses were performed at day 30 and day 90: before being placed in MRI devices, all animals were sedated. At day 90, after all imaging analyses were performed, animals were euthanised. The subcutaneous plane was initially exposed and the scar tissue over the mesh was examined. The inner surface of the mesh was then inspected via a U-shaped incision. None of the animals died during surgery. Lastly 12 rats, in which no mesh was implanted, were used as negative controls for gadolinium toxicity measures (experimental group VI). Rats were kept isolated for 3 days after operation then caged in pairs. Analgesic treatment was applied routinely once a day for 3 days after surgery by subcutaneous injection of Metacam 0.07 ml per 400 g bodyweight, as described before [12]. Death and presence of clinical signs of disease (weight, fur, presence if stools, signs of prostration and aggressiveness) were monitored daily by the animal housing operators and weekly by the veterinary surgeon.

Gd-PCL and Gd-PMA were coated onto PP mesh (manufacturer COVIDIEN, Trévoux, France) as described before [17].

### Histology and immunohistochemistry

Thirty days after surgery (D = 30) prostheses were explanted and the entire anterior abdominal wall (from the skin until the peritoneum) was harvested. For each test group (Gd-PMA, Gd-PCL and control) three biopsies were collected. Each specimen was divided into two parts along the longitudinal axis (middle line Fig. 2C) in order to have specimens for histopathological and specimens for immunohistochemical analyses. For standard histological analysis, samples were fixed in formaldehyde (5%), dehydrated with double alcohol and toluene substitution, five tissue sections of 4 μm were collected for each sample (cut adjacently) and stained with Hematoxyline Eosine and Safran (HES) coloration. Immunohistochemistry for types I and III collagen was also performed: specimens were frozen in liquid nitrogen and then stored at −80°C, five slices of 10 μm (cut adjacently) were obtained at the cryomicrotome and then fixed in acetone. Analysis of extracellular matrix was carried out by labelling type I and III collagens with rabbit anti-rat collagen I (abcam #ab24133) at 1:250 dilutions and rabbit anti-rat collagen III (abcam #ab7778) at 1:200 dilutions. For the analysis of vascularisation smooth muscle α-actin was labelled with anti-rat antibody (abcam #ab5694) at 1:200 dilution. The secondary antibodies used were peroxidase-conjugated Affinipure Goat anti-rabbit (111–035–144); peroxidase-conjugated Affinipure Goat anti-mouse (115–035–146) (both purchased from Jackson Immuno Research).

All histology and immunohistochemistry analyses were performed with a Leica Optical Microscope at 40X magnification.

### MR imaging

We sought to optimise the MRI sequences to visualise the polymer-coated mesh. The objective was to obtain a T1- weighted image. As first step, experimental (7T) MRI (Bruker Avance DRX system) was used to obtain high resolution 3D images of Gd-PCL and Gd-PMA coated meshes. Images were acquired with standard T1-weighted fast spoiled gradient recalled echo (FSPGR) [17].

To define and optimise sequences for visualisation of meshes *in vivo*, coated implants were embedded in 1% w/w low gelling point agarose type VII (Sigma Aldrich, France) or inserted in muscle tissue within 20mL disposable scintillation glass vials (Dutcher, France) and then placed in 3T MRI (GE Healthcare) devices for analysis. Gradient echo sequence, routinely used in clinical practice for the exploration of the pelvis, was used. In order to assure reproducibility, the best sequences obtained for both 3T and 7T (3D T1-weighted spoiled gradient echo images were acquired with TR/TE = 100/3ms, alpha = 75°, a field-of-view of 30x30x10 mm3 and a matrix of 64x64x32 matrix. Two averages were performed leading to an imaging time of 11 min) were used to acquire all images. To verify the stability of the coated meshes, samples were stored up to one year at 37°C and tested for imaging at 4 months and one year, the size of the mesh was measured at every imaging assay. The reconstruction of images obtained was performed using the 3D reconstruction software OsiriX.

### Toxicity measures

The validation of the stability of the Gd-polymers was confirmed by assaying the diffusion of gadolinium by inductively coupled plasma mass spectrometry (ICP-MS) [22,23]. The ICP-MS allows us to measure the amount of gadolinium in organs after mesh implantation. Peripheral and systemic distribution of gadolinium toxicity was assessed in implanted rats and compared to control group (at D90). To detect gadolinium accumulation in organs: liver and kidney tissues were harvested and processed. A few milligrams of the samples were weighed and dissolved with concentrated nitric acid in suprapure quality (Merck, Darmstadt, Germany) in a glass tube at 80°C. For calibration, a gadolinium standard solution was diluted with deionized water to obtain calibration solutions. ICP-MS operating conditions were optimized to obtain the highest signal-to-background ratio for gadolinium, the isotope that was applied for the analytical determination.

### Statistical Analyses

The number of animals was based on published studies [24,25]. All statistical analyses were performed with SAS (version 8) statistical software. Data are expressed as mean ± standard deviations (SD). Statistical significance of differences among groups (implanted vs control groups) was evaluated with a t-test with level of significance set at P<0.05.

## Results

### Clinical Tolerance of Gd-Polymers coated mesh

The evaluation of the clinical tolerance on an animal model and the non-systemic diffusion of Gadolinium are essential features to meet the pre-clinical characteristics of the implant.

**Fig 3:**
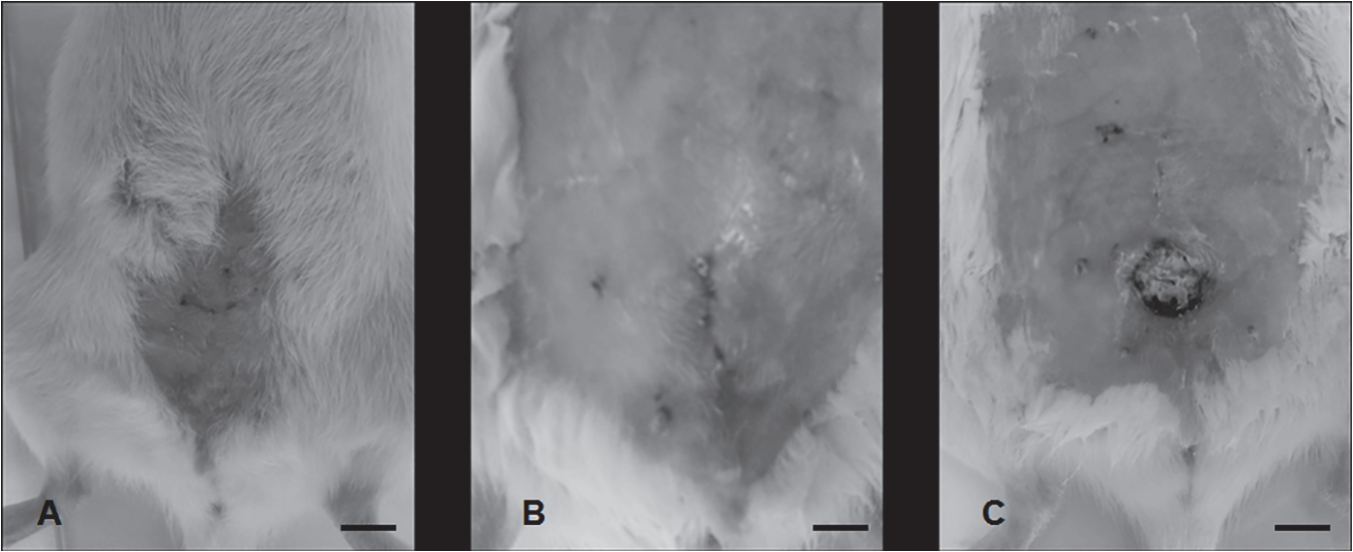
Postoperative inspection of the rats. Clinical observation at day 30. Representative images of (A) Non-coated PP mesh control group (III); (B) Gd-PMA coated mesh experimental group (I) and (C) Gd-PCL coated mesh experimental group (II). Erosion was visible only on Gd-PCL implanted group. Scale bars: (A) 1cm, (B) 2mm an (C) 0,8cm.

At three months after implantation, the healing process was completely achieved for the animals implanted with Gd-PMA and PMA/PMMA meshes while rats implanted with Gd-PCL meshes displayed a noticeable erosion rate (100% (n = 10/10) Gd-PCL vs 0% (n = 0/10) Gd-PMA) as shown in Fig. 3. Mean apparition of the erosion on PCL meshes was at 21±3 days.

**Fig 4:**
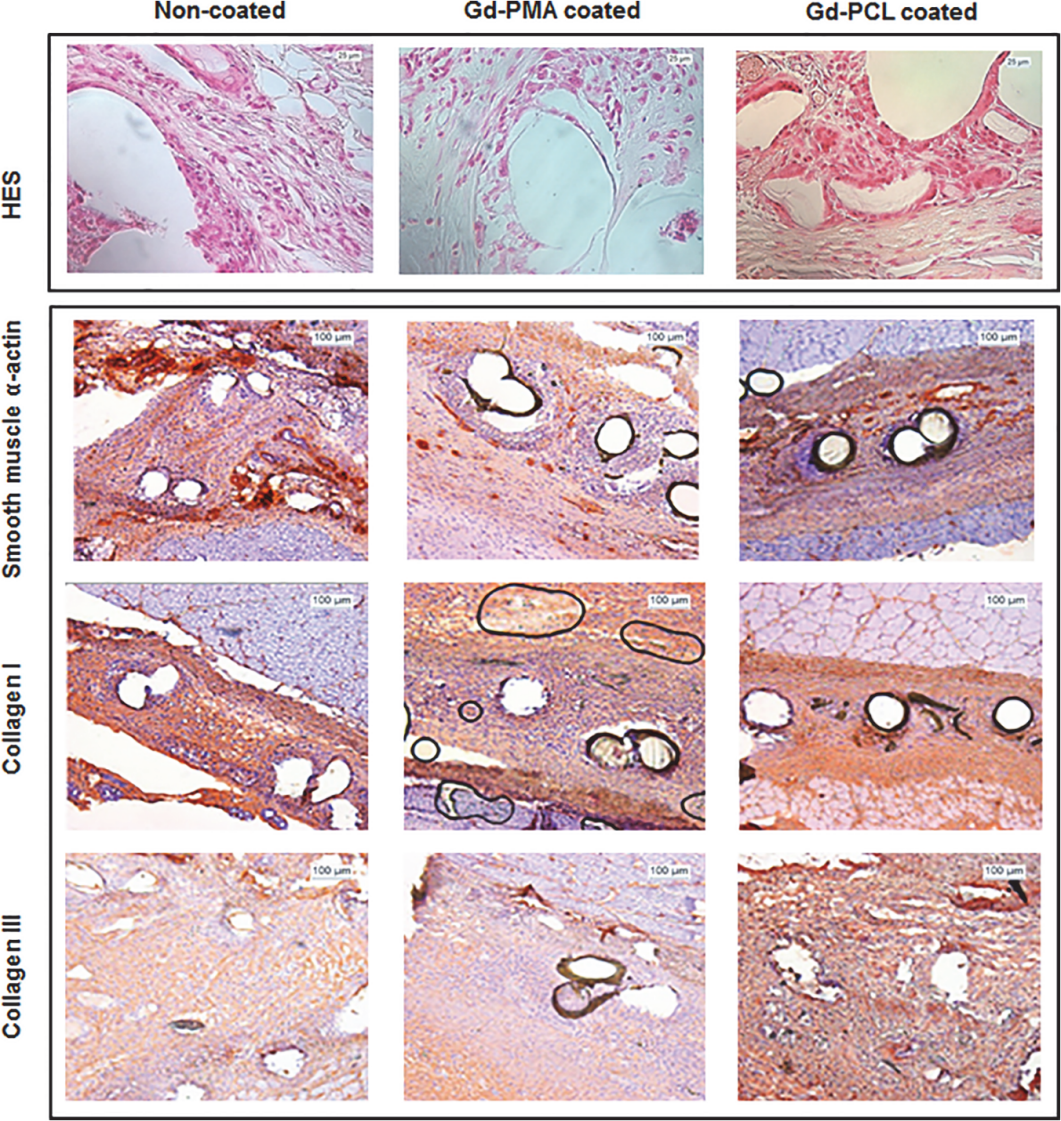
Histological images of the explanted meshes. Upper panel: Standard histological results at day 30 of Hematoxyline Eosine and Safran (HES) stained sections of non-coated mesh (control group I), Gd-PMA coated mesh (experimental group II) and Gd-PCL coated mesh (experimental group III). Lower panel: Immuno-histochemistry sections of non-coated mesh (control group I), Gd-PMA coated mesh (experimental group II) and Gd-PCL coated mesh (experimental group III) labelled for smooth muscle α-actin, collagen I and collagen type III (Optical microscope X40 magnification).

Microscopic analysis of histology sections showed mild inflammation for the non-coated mesh and Gd-PMA coated mesh groups. For both these groups the tissue surrounding the implants was characterised by the presence of non-inflammatory collagen and important blood supply. For the Gd-PMA group in particular, substantial neoangiogenesis with capillaries and venules could be observed. For the non-coated mesh group, the tissue was also characterised by the presence of myofibroblasts with good mesenchymal organisation. On the other hand tissues surrounding Gd-PCL coated mesh were characterised by the presence of disordered collagen, persistent cellular inflammation and giant cell granulomas. Also, low levels of neo angiogenesis were visible (Fig. 4).

Immunohistochemistry showed similar patterns of smooth muscle α-actin, types I and III collagen between non-coated and Gd-PMA coated mesh groups. In particular, for the non-coated mesh group we could observe well organised blood vessels, which were no longer capillaries but mainly venules and arterioles. For the Gd-PMA group blood vessels were organised in an intermediary structure (few capillaries and mostly venules and arterioles). Tissues from Gd-PCL implanted rats show a high level of inflammation with few blood vessels mainly capillaries. Types I and III staining showed a good extracellular matrix organisation for the uncoated and Gd-PMA coated implants with a higher amount of collagen I compared to collagen III. For the Gd-PCL on the contrary, very low levels of collagen I were visible (Fig. 4).

### *In vivo* imaging

For imaging of the coated meshes both experimental 7T and clinical 3T were used. Due to the very poor tolerance of Gd-PCL meshes, animals implanted with these implants were excluded from imaging assays.

With both techniques we obtained a clear visualisation of a Gd-PMA mesh coated at a dose of 3.8 μg / cm^2^. Coated meshes were visible with both 3T and 7T MRI techniques. All meshes were entirely a homogenously visible; indeed, the size of the mesh (image) corresponded to the size of the mesh *in vivo*.

**Fig 5:**
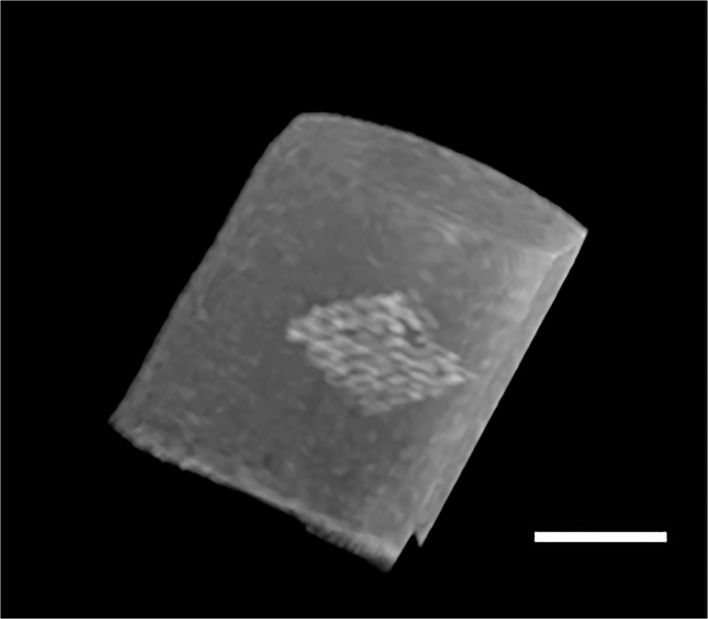
MRI images of the mesh in vitro. Gd-PMA coated mesh with a 3T MRI device, reconstruction was obtained with osiriX software. Scale bar 1 cm.

**Fig 6:**
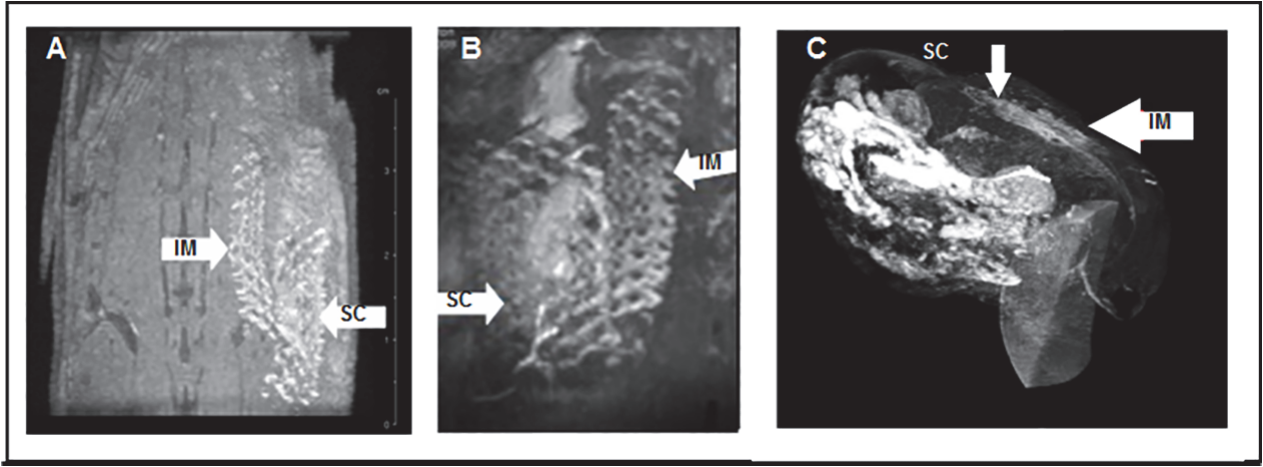
In vivo visualisation of the mesh. **(A)** Frontal and **(B**) lateral view of Gd-PMA coated meshes as seen with a 7T MRI device, 3D reconstruction was obtained with OsiriX software. **(C)** 3D reconstruction of a Gd-PMA coated mesh (dorsal implantation) (3T MRI). Note MRI imaging and 3D reconstruction allow us to distinguish between two meshes (arrows) implanted subcutaneously (SC) and intramuscularly (IM) within few millimetres distance. Scale bars in A and C 1cm, B 5mm.

3D reconstruction program allowed the optimisation of MR imaging by highlighting the contrast of the mesh and therefore allowing us to distinguish the implant within the different muscular layers (Figs. 5 and 6).

### *In vivo* toxicity evaluation

The ICP-MS evaluation after degradation of explanted meshes allowed to measure residual doses of gadolinium in surrounding tissues and in the organs metabolising Gd. The concentrations were measured in liver, kidney, tibia bone and skin of the animals implanted with coated meshes. No significant differences in mean levels of gadolinium were found in implanted group as compared to control group (Table 1).

**Table 1:** Gadolinium concentrations (ppm) measured in rats implanted with coated meshes (implanted group) and not-implanted (control group).

Organs	Implanted group (n = 12)	Control group (n = 12)
**Liver**	0.17±0.08	0.17±0.02
**Kidney**	0.09±0.02	0.07±0.02
**Tibia bone**	0.05±0.02	0.12±0.03
**Skin**	0.09±0.02	0.09±0.01

## Discussion

With this study we sought to evaluate the tolerance and long-term MRI visibility of Gadolinium-modified polypropylene meshes for their use in soft tissue repair.

Appropriate modification of the meshes used for reinforcing the tissues in pelvic organ disorders enables their visualisation on magnetic resonance imaging [17]. This technology may represent an essential feature to optimise the management of complications and the recurrence of soft tissue and therefore its surgical management. The follow-up by ultrasound techniques has been previously investigated but their use has been restricted to the visualisation of meshes only in the vaginal segment. At the same time MRI has emerged as the tool of choice for monitoring implantation of meshes in the tissues [26–28]. Hansen and colleagues showed that iron particles incorporated into polymer-based implants allow MRI visualisation and offer a non-invasive alternative to surgical exploration in case of suspected mesh-related complications [27]. The exploration of the pelvis by MRI provides a lot of useful information for the pre and postoperative monitoring of all gynaecological pathologies requiring surgical management. Initially used for neuroradiology applications, 3T high-field MRI was quickly adopted in many scientific and clinical domains such as uro-gynaecology [29]. Previous studies showed that MRI-visible polymer (DTPA-Gd-PCL) coated onto PP meshes enables *in vitro* visualisation of the mesh for at least one year [30]. Also, Gd-PMA coated meshes revealed good level of cytocompatibility [14]. With this study we aimed to compare both polymer (PMA and PCL) not only in terms of in vivo long-term MRI-visibility but also in terms of biocompatibility and in vivo tolerance. Interestingly, in vitro results, which suggested PCL as equally good polymer for gadolinium linking to a PP mesh, were not supported by in vivo experiments and clinical observations. A possible explanation of high level of erosion observed in Gd-PCL implanted rats may be that these polymers release acid products upon biodegradation that may lead to inflammatory reactions and eventually tissue necrosis [31]. In this study we showed that Gd-PMA does not provoke any erosion, allows a good integration of the implant with low levels of inflammation, a suitable extracellular matrix organisation and vascularisation of the integration tissues. A limitation of this study is that only visual inspection was performed on the HES and IHC sections without a precise scoring. However, as the erosion provoked by of the Gd-PCL implants was observed before the histological analyses could be performed; the scoring was not considered to add valuable information. All together these observations suggest that PMA is a linking polymer suitable for implantable devices modification. PP was chosen as standard support material, but Gd-PMA linking technique may as well be extended to other biomaterials used to synthesise meshes.

Gadolinium was not the only MRI contrast agent that may have been selected for this purpose. Fluorinated derivatives have been exploited before [32], but resulting images consisted only in a hyposignal therefore reducing the interest in pelvis MR imaging.

The MRI visualisation was obtained in vitro for both types of polymers (Gd-PCL and Gd-PMA), but the intensity of the signal observed was stronger with PMA than with PCL, probably because of its hydrophilic nature. As discussed above, the cutaneous and tissular integration was not clinically satisfactory in the case of the Gd-PCL implants confirming that Gd-PMA were more suitable for further investigation.

The amount of gadolinium necessary for mesh imaging is correlated to the type of MRI, the tissues surrounding the area of the implants and their intrinsic contrasts. Results from previous studies helped to reinforce the idea that low doses of gadolinium are necessary (ranging from 1 to 20μg / cm2 according to the type of MRI used) [29].

*In vivo* MR imaging raises the issue of the implant precise location and the risk of confusion with many artefacts due to the production of millimetre slices. The imaging artefacts, such as respiratory movements of animals, may hamper the image optimisation in abdominal position. On the contrary, the dorsal implantation of the mesh allowed us to precisely locate two very close (mm distance) implants. Nevertheless, these distances may entail more artefacts; discrimination of such artefact was however confirmed by accurate 3D images reconstruction (Fig. 6).

Experimental studies on the rat model requires many volume settings directly related to the size of the animal and therefore to the sample. For instance, standard settings in clinical MRI allow us to obtain cuts of 5mm. Because of the millimetre range of meshes it was necessary to reduce the thickness to 3 mm as well as to adapt the sequences. Pre-sets and standardised parameters in 3T MRI adapted to clinical situations do not exist on 7T MRI where each parameter is defined independently. It is therefore difficult to establish correspondences between clinical and experimental MRI. Another limitation of this study is represented by the animal model itself: the time period during which rats may be followed is limited to three months, which does not allow extrapolation to humans and conclusion about the long-term (years) effects of the implants. This problem may be only partially overcome by testing the Gd-modified meshes on a bigger (ovine) model which allow a longer follow-up period.

Nephrogenic systemic fibrosis (NSF) induced by Gadolinium-based contrast agent, has been described in rats, mainly in the dermic tissue [22]. Hence, it is important to be able to determine the presence of free Gadolinium in tissues immediately adjacent to the implanted mesh, in skin and various organs (liver and kidney) [22]. ICP-MS is a type of mass spectrometry capable of detecting very low concentrations of metal with greater precision and sensitivity as compared to spectroscopic techniques [23]. ICP-MS assays performed on tissues harvested from implanted animals, have highlighted the absence of diffusion of Gadolinium and therefore minimal risk of nephrogenic systemic fibrosis.

The last decades have seen a remarkable growth in the applications of MR imaging. The need for the visualisation of meshes used in pelvic floor surgery derives directly from the necessity to optimise the monitoring and care of patients. The stability of the Gd-complex coated meshes allows the visualisation over time of the implants. By linking the clinical symptoms to the observation of an anatomical region will result in the improved management of complications. Monitoring of the mesh would allow clinicians to assign responsibility for a post-operative symptom. In conclusion, the technique described above allows a non-invasive and long-term imaging of implants.
